# *Polisye Kont Moustik*: A Culturally Competent Approach to Larval Source Reduction in the Context of Lymphatic Filariasis and Malaria Elimination in Haiti

**DOI:** 10.3390/tropicalmed2030039

**Published:** 2017-08-18

**Authors:** Kevin Louis Bardosh, Lorence Jean, Valery Madsen Beau De Rochars, Jean Frantz Lemoine, Bernard Okech, Sadie Jane Ryan, Sue Welburn, J. Glenn Morris

**Affiliations:** 1 Department of Anthropology, University of Florida, Gainesville, FL 32611, USA; 2 Emerging Pathogens Institute, University of Florida, Gainesville, FL 32611, USA; malolo2001@yahoo.com (L.J.); madsenbeau@phhp.ufl.edu (V.M.B.D.R.); bokech@ufl.edu (B.O.); sjryan@ufl.edu (S.J.R.); jgmorris@epi.ufl.edu (J.G.M.J.); 3 Ministry of Public Health and Population, Cap-Haitien, Haiti; 4 Department of Health Services Research, Management, and Policy, University of Florida, Gainesville, FL 32611, USA; 5 Ministry of Public Health and Population, Port-au-Prince, Haiti; tileum@hotmail.com; 6 Department of Environmental and Global Health, University of Florida, Gainesville, FL 32611, USA; 7 Department of Geography, University of Florida, Gainesville, FL 32611, USA; 8 School of Biomedical Sciences, Edinburgh Medical School, The University of Edinburgh, Edinburgh EH8 9YL, UK; Sue.Welburn@ed.ac.uk; 9 Department of Medicine, University of Florida, Gainesville, FL 32611, USA

**Keywords:** vector control, mosquitoes, community-based, participation, social science, malaria, lymphatic filariasis, Haiti

## Abstract

Community engagement has become an increasingly important focus of global health programs. Arbovirus emergence in the Americas (Zika and chikungunya virues), and global goals for malaria and lymphatic filariasis elimination, mean that community-based mosquito control has taken on a new salience. But how should mosquito control initiatives be designed and implemented in ways that best engage local people? What are the challenges and trade-offs of different strategies, not only for effectiveness but also for scale-up? In this paper, we describe the social and political dynamics of a pilot study in a small town in northern Haiti. With the aim of developing a culturally-competent approach to larval source management (LSM), our pilot project combined larval surveillance with environmental management, social engagement, community education, and larvicide application. Orientated around a network of ‘Mosquito Police’ (*Polisye Kont Moustik*, in Haitian Creole), our approach integrated elements of formative research, social learning, and community participation. Here, we reflect on the challenges we encountered in the field, from larval mapping, staff management, education and behavior change, engagement with formal and informal leaders, and community-based environmental cleanup. We discuss how these programmatic efforts were influenced and shaped by a complex range of social, cultural, political, and economic realities, and conclude by discussing the implications of our community-based approach for the elimination of lymphatic filariasis and malaria, and other vector-borne diseases, in Haiti.

## 1. Introduction

Policy and discourse in global health continues to stress the importance of community engagement and social mobilization. Responses to Ebola in West Africa, polio eradication in Central Asia, and Zika virus in the Americas have all emphasized the need for new approaches in research, surveillance, response, and prevention. However, this rhetorical normalization hides a more challenging reality: we continue to struggle with the creation of scalable and culturally-competent approaches to engage local people in public health, especially in the social determinants of health [1,2,3]. Competing notions of what participation means, what it entails in practical terms, and how (cost) effectively it can achieve health goals are at the center of fluctuating ideological and financial support. The rhetoric significantly lags behind tangible investments by major donors, agencies, and governments. Decade-old tensions between vertical and horizontal approaches also continue to be widely debated, with limited progress [4].

Larval source management (LSM)—the control of aquatic mosquito habitats responsible for breeding disease-transmitting mosquitoes—is one area where community participation has been widely emphasized but selectively implemented or simply overlooked [5]. Involving local people in environmental sanitation is not only a matter of individual and household behavior change in garbage disposal, water storage, and hygiene. It also involves wider systems of governance and infrastructure that surround larval habitats, and the economic, political, and social contexts that accompany them. In this paper, we explore these issues, focused on mosquito-borne diseases in Haiti. 

LSM has been central to *Aedes aegypti* control, responsible for transmitting dengue, chikungunya, yellow fever, and now Zika. *Aedes* mosquitoes are container breeders, well adapted to the urban built environment especially the urbanizing cities and slums of tropical and sub-tropical regions [6]. A range of social mobilization and engagement approaches have been trialed: interventions to address water storage (through container lids and covers), cleanup campaigns to reduce potential man-made habitats, health communication to raise awareness, and larvicide-based treatment of household water containers [7,8,9,10,11]. Efforts at institutionalization, however, have been challenging outside of major epidemics. 

Traction for LSM has been much slower for malaria and lymphatic filariasis (LF), where policy is centered on targeting the pathogen (rapid tests and treatments for malaria and mass drug administration (MDA) for LF) and the adult mosquito (long-lasting impregnated nets (LLINs) and indoor residual spraying (IRS)). Larviciding and environmental management campaigns of the early 20th century, before the advent of DDT, were hugely successful in controlling malaria [12,13]. While malaria burden worldwide has recently reduced [14], challenges of pyrethroid resistance and the need to target outdoor transmission makes LSM an appealing approach. Studies in Burkina Faso [15] and Tanzania [16] have shown promising results using a biological larvicide, *Bacillus thuringiensis israelensis* (*Bti*) [17,18]. LSM remains the core focus of mosquito control programs in developed countries; mosquito abatement districts in the United States and throughout Europe are centered on LSM, funded by municipal taxes, for the control of nuisance mosquitoes [12].

Similar arguments can be made for LF. Treatment coverage for the Global Programme to Eliminate Lymphatic Filariasis (GPELF), launched in 2000 with the goal of elimination by 2020, has been successful in achieving high levels of annual MDA coverage (>65%), needing over 4–6 years to effectively interrupt transmission [19]. In some foci, this has not proved sufficient: eco-epidemiological and genetic factors, uncertainty about the level of microfilarial suppression needed, and persistent ‘non-compliers’ are involved [20,21,22]. In Haiti, *Culex quinquefasciatus* breeds in polluted and stagnant water accumulating in urban and peri-urban areas, where garbage collection and urban planning are compromised. Sanitation campaigns have been found to be effective at reducing LF in certain foci, and could be integrated into MDA [23].

In many foci, some of these different diseases are transmitted either by the same mosquito species or occur in similar eco-epidemiological zones, making integrated vector management (IVM) an appealing approach to optimize resources and improve poor environmental sanitation conditions [24]. Community-based environmental management in drains, ditches, and canals, has shown promise but studies have been very limited [25]. Community-directed LSM is not a panacea. However,it has been under-utilized and should be re-examined in the context of malaria and lymphatic filariasis elimination targets, the so-called ‘endgame’ stage [26], and because of the continued emergence of new arboviruses [27]. A number of persistent misconceptions and myths, including doubts about evidence and efficacy, feasibility in resource-poor settings, and concerns about management and costs, need to be more systematically addressed to build the evidence-base for LSM (see [18]). 

In this paper, we reflect on our experience in designing and implementing a community-directed LSM initiative in a small town in northern Haiti, an area endemic for multiple mosquito-borne diseases: lymphatic filariasis, malaria, Zika, chikungunya, and dengue. Our demonstration project—*Polisye Kont Moustik*, or Mosquito Police—aimed to develop a culturally-competent approach to environmental sanitation, vector control, and social engagement in the Haitian context.

Haiti has some of the worst health indicators globally; the health delivery landscape is fragmented, dominated by NGOs and heavily reliant on international aid. Total expenditure on health per capita was $133 US dollars in 2014, according to the WHO (http://www.who.int/countries/hti/en/). Cycles of foreign intervention, the lingering effects of political instability and economic underdevelopment, and the legacy of natural disasters continue to betray efforts to improve this [28,29,30]. During our pilot project, the United Nations Stabilization Mission in Haiti (MINUSTAH) was over a decade old, a contested national election ushered in an uncertain transitional government, public hospitals were on strike, Hurricane Matthew devastated the southern coast, and the infamous cholera epidemic—imported by Nepali UN troops in late 2010—continued in the face of meager international donations.

Malaria and LF have been widespread for over 250 years in Haiti, having arrived with slave ships from West Africa. The first nation-wide LF survey showed a 7.3% filarial antigenemia, by far the largest prevalence in the Americas [31]. Since then, a highly successful national MDA campaign has distributed annual DEC and albendazole treatments, using community-based agents and directly observed treatment at established distribution posts [32]. The program reached full national scale in 2012, although a number of persistent hotspots (including our study site) show persistent LF transmission despite 10 years of MDA. Malaria also remains endemic in Haiti, one of the last areas in the Americas, mostly caused by *Plasmodium falciparum*. The Malaria Zero Campaign aims to eliminate malaria by 2020. The argument for elimination in such a short-time period is based on the fact that *P. falciparum* exists at <1% at population level, there is no resistance to chloroquine, there is little malaria importation, and *Anopheles albimanus* is a poor vector (predominately feeding outdoors and on animals).

In this paper, we explore the social and political dynamics of a community-directed LSM pilot project in northern Haiti using an anthropological approach. We present an informed ethnographic analysis of the micro-politics of design, context, implementation, and transformation as we sought to engage district officials, municipal authorities, local gatekeepers, our staff and community members in LSM. Such a ‘messy analysis’, so we argue, is fundamental to understanding why, how, and when social engagement and community education approaches can work—or fail to work—for the prevention of mosquito-borne illnesses in Haiti, and elsewhere. 

## 2. Methods and Study Site

The goal of the *Polisye Kont Moustik* (PKM) project was to develop and validate a community-directed approach to LSM that could (in one form or another) be integrated and scaled-up with existing efforts to eliminate malaria and lymphatic filariasis by 2020 in Haiti, specifically in persistent hotspot foci. An action research project, PKM was implemented over 20-months between September 2015 and April 2017. Our study was approved both by the IRB committee of the University of Florida and the National Bioethics Committee of the Ministry of Health, Haiti. 

A first research phase (September–December 2015) involved a scoping analysis (25 key informant interviews) of vector control activities in Gressier, Leogane, Cap-Haitien, and Port-au-Prince, with NGOs, government, and international research partners. This analysis lead us to identify community-directed LSM as an area where further evidence was needed, and the Cap-Haitien area (Haiti’s second largest urban area) as an appropriate study location. A second research phase involved discussions with MSPP (Ministry of Public Health and Population) staff for the selection of study sites in the North Department and the recruitment of staff, including a research coordinator (co-author LJ), and the generation of baseline data. An initial formative research phase involved a household questionnaire (HHQ) on environmental sanitation (*n =* 293), focus group discussions (6 FGDs with community leaders), and key informant interviews with formal and informal local leaders and MSPP staff (*n =* 17). The HHQ explored local knowledge, attitudes, and practices related to mosquito-borne diseases, diarrhea, cholera, and helminth infections. Informal interviews and participant observation continued throughout the project. 

Our study sites were divided into four intervention *quartiers* and four control *quartiers* (eight total sites), with roughly 2500 household and 10,000 people, in order to evaluate the impact of our LSM activities. Our four intervention *quartiers* comprised the geographically continuous small town of Plaine-du-Nord, an important voodoo pilgrimage site surrounded by fertile agricultural land and an interesting history going back to the early Haitian revolution. During the nationwide LF prevalence survey conducted in 2000s, Plaine-du-Nord had a staggering 45% filarial antigenemia prevalence, the highest prevalence in Haiti [31]. It is a small town of 1181 houses and 4257 people (according to a household census we conducted), with electricity, a small river and system of cement/soil canals. Our four control *quartiers* (1328 households and 4984 people) were selected in areas of Plaine-du-Nord, Milot, and Lemonade commune to reflect different socio-ecological conditions, including a dense urban area, a very rural area with large tracks of farmland, and two peri-urban *quartiers*.

From April 2016 to April 2017, we conducted a series of interventions designed, in a stepwise approach, to test different community mobilization strategies, larval surveillance techniques, community education and community-directed LSM, including the use of larvicide and environmental modification. These were conducted in tandem with periodic meetings, using participatory rural appraisal techniques (PRA), with a wide range of local religious, school, municipal and community group leaders, who were directly involved in the project. It also included monthly reflection workshops with our project staff, allowing us to problem solve in the field. 

To better contextualize our analysis and understand the potential to scale-up, between October 2016 and February 2017, community participants in Plaine-du-Nord (17 women and 15 men, aged between 18–90 years) were selected from different social and economic strata for in-depth interviews. The purpose of these interviews was to better understand local perceptions of disease and the social and cultural context of participation in the project. These interviews were conducted with local leaders (municipal leaders, religious leaders, school teachers, health workers), street cleaners, market sellers, poorer agricultural laborers, woman-led families, and middle-income families. 

Data presented in this article also reflect one-year of ethnographic data compiled by the principal investigator (K.L.B.), a medical anthropologist, on the cognitive and social dimensions of implementation. This data reflected on the challenges we encountered in the field and the decisions we made, and relied on a combination of detailed fieldnotes, participant observation, and unstructured interviews. Where appropriate, we further describe our methods in the text below. 

## 3. Results

### 3.1. Developing a Mapping and Management System

The foundation of LSM is surveillance; the need to locate, evaluate, and manage larval habitats and productivity is paramount, albeit limited entomological data presents a challenge in Haiti [33]. Large swaths of territory need to be reduced into the ‘few’, ‘fixed’, and ‘findable’ habitats that should be prioritized. To do so, LSM relies on a strong field management system—the militant historic examples of Fred Soper and William Gorgas are frequently mentioned [12]. The logistical complexities of mapping and management are often invoked to explain-away the feasibility of LSM in places like Haiti. Hence, it was apparent from the beginning of our project that developing a culturally competent management system would be key. This was not an easy process and required trial, error, and a number of different ‘systems’. 

The first involved the recruitment of eight ‘mosquito police’ and two supervisors across the eight *quartiers*. Our aim was to employ these local people to balance the demands of weekly larval surveillance, community engagement, and larvicide application. Our initial recruitment process, in early 2016, involved consulting local political and civic leaders and obtaining their recommendations. We then interviewed these individuals, selected the best for our first training and began developing a geospatial system to follow all larval habitats over time. To do this, we created a unique identifier system, following the methodology outlined in Fillinger et al. [16] and Dongus et al. [34] from the Urban Malaria Control Programme in Dar es Salaam, Tanzania. The Tanzanian system relied on existing political administrative units, including the segregation of wards, neighborhoods, and ‘10-cell-units’ (TCUs), the smallest administrative unit in Tanzania (typically comprising 10 houses and headed by an elected chairperson). No such system exists in Haiti, where leadership, community, and geographical structures are more fluid. Rather, we had to create our own segregations, which required considerable time and effort in cartography.

Our mapping system avoided the use of physical identifiers on houses or public spaces. Any labeling system would be viewed as a precursor to taxation, political manipulation, or both, and would be resisted, so we were told. Instead, we combined the use of Google Earth maps of each of the eight *quartiers*, printed, and glued onto laminated poster-boards so that they could be carried in the field, with hand-drawn field maps. Following the Tanzanian project, we divided each *quartier* into ‘blocks’ and each block into approximately 10 ‘places’, which included one or two houses and the surrounding road, vegetation, and yard. Maps of each block were drawn by the eight mosquito police working in teams of two to ensure accuracy and then arranged into a laminated field-book. The combined training and inventory of places took nearly one month, as some areas had to be re-done after quality checks and rainy weather. Our system included 319 ‘blocks’ and 3567 distinct ‘places’ across the eight sites (to be surveyed weekly), nearly equal between interventions (160 blocks and 1765 places) and control *quartiers* (159 blocks and 1802 places) (Table 1). According to a household census we conducted, this included 2509 houses and 9241 people. 

After mapping, we conducted one-month of baseline larval surveillance, which we planned to use in evaluating community-based control activities. We stressed the need to ‘think like a policeman’*—*be systematic, precise, focused, hardworking, punctual, and approachable to the community. At the end of this month, community leaders accompanied PKMs on their rounds to increase local understanding of larval habitats, mosquito ecology, and to brainstorm strategies for environmental cleanup and modification approaches. With more attentive supervision, however, the number of potential and positive larval habitats more than doubled, revealing some problems. First, we had under-estimated the number of houses, meaning that the ratio of PKMs to households was unrealistic; field staff were stretched beyond capacity. Our desire to minimize the number of PKMs to keep labor costs as low as possible, motivated by our future consideration for scaling up, meant that the quality of inspections (especially household visits to cover the container-breeding *Aedes* mosquito) was compromised. Second, it was clear that about half of our initial staff were not well-suited to the job, a common problem with community health workers [35]. Without a physical identifier system, we had inadvertently consolidated power into the hands of each individual PKM, making it tricky to let people go who tried to “dodge the work, and obtain the salary without really working” (interview). Despite being selected by local leaders, complaints were voiced that some staff lacked the authority and/or confidence to conduct house inspections and successfully engage community members in environmental management. Lastly, we found that one of our field supervisors and our quality controller had been misreporting the work schedules of the PKMs in the field and quality control checks. This was described as a form of “sabotage…they entered into complicity with each other. They made a pact to not work too hard and to lie for each other about how the work was going.” (Interview.) Other members of the team had been reluctant to report this. As one our Ministry of Health partners discussed with us:

“Not everyone can manage people or speak to them in persuading ways to instill a sense of teamwork. You need orientation in community work. You need to understand people’s interests and move together…It is hard (for the PKMs) to point the finger at people in [their] community, especially when they have more power than you. In Haiti, the biggest problem is getting people to move with you and to be honest in their work.”

**Figure 1:**
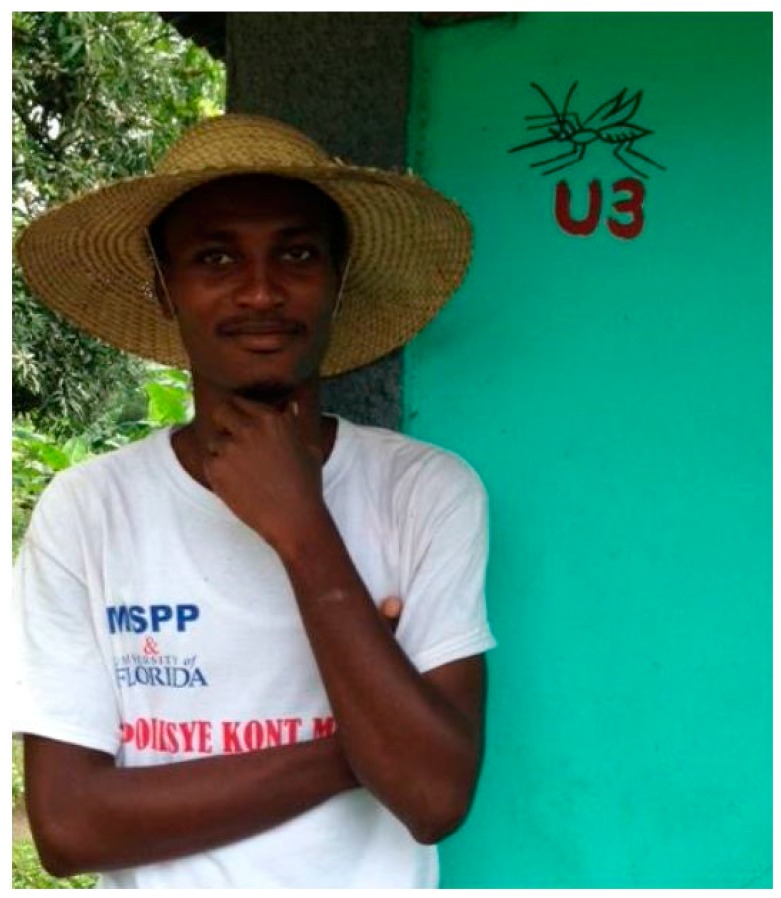
A PKM with an example of our mosquito code in the background.

To develop a stronger staff management system, we implemented a number of improvements. Through discussions with community members, we found that a physical identifier system was acceptable, partially due to the wide sensitization efforts we had done: we had effectively built a sense of trust with the community (see details below). To avoid any suspicions, local leaders recommended that the identifier system include a picture of a mosquito with letters and numbers (See Figure 1). This increased our ability to do random field checks and monitor fieldwork. Through our community-based activities we also had come to identify key individuals who better fit our job requirements. It became apparent that youth (men and women) and peasant farmers were the individuals most able and willing to be employed for LSM, but were barred by local elites in access to employment opportunities. To address the challenges of covering 3567 unique places, and between 2000 and 3000 potential habitats each week, we increased our staff from 8 to 20 PKMs, and from 2 to 4 supervisors. We ensured that quality checks were done systematically, with each supervisor working in a different *quartier* each week, in rotation. After each quality check, any differences between surveillance sheets were discussed in a weekly Friday meeting with all staff members. Lastly, we also added an effort-based salary bonus, with morning and afternoon sign-in sheets and random spot checks by the research manager. 

One issue that required a different sort of solution involved our recruitment process. Clearly, selecting individuals solely from the recommendations of local leaders left the situation open to nepotism. We decided to do two things: to train a lot more people than we needed and to create practical and theory tests to vet recommended individuals. The test proved critical in circumventing the otherwise problematic and strong pressure to select individuals, promoted by key local formal and informal leaders, that were either not competent and/or not interested in working (but still wanted to obtain the salary), as well as a general sense of fairness (that recruitment was not being dominated by a small number of people).

All of these efforts served to increase our ability to manage our staff, placing them ‘under control’ so that we could ‘stay with them’ through relational bonds and social learning. A senior MSPP official who visited our project area stated: “It is amazing that you have local people working so hard! I have never seen this before. Even in the city, I cannot get people [at MSPP] to work past 1p.m. in the office” (interview). The fact that staff were working from 8 a.m. to 4 p.m. each day, in the heat and sun, was an admirable achievement—genuine interest in the goals of the project, a sense of teamwork and motivation to show that community-directed LSM could make a difference in the community were important.

“Leadership needs to start at the top. Field workers need to see that their bosses are also working hard and not just exploiting them, and gaining from them. This creates respect and this is the foundation of the problem of governance in (Haiti)…the leaders are not modeling ethics for people. So that is really an important part of this project.”

(Interview, 2016.)

In de-brief meetings, our team continuously drew on local idioms to explain how project work evolved in Haiti: you had to ‘use tactics to ‘avoid sabotage’ and ‘build confidence’ so that people would ‘go with you’. These terms became especially important as we negotiated and navigated the local political context. 

### 3.2. Engaging Municipal Leaders During a ‘Crisis of Confidence’

A central mantra in global health is the need to engage local leaders. Socio-political relationships and histories created unique tensions in Haiti; widespread disenchantment with local political elites, frequently discussed as ‘broken promises that are never kept’, underpinned what was widely discussed as a deeply-rooted ‘crisis of confidence’, one that influenced our efforts to engage community members in LSM.

Our first meeting with the mayors of Plaine-du-Nord town was positive. Newly elected after four years of rule by another political party, the new leadership eagerly accepted a project that would help them with environmental sanitation. By linking existing municipal waste collection with larval control, and working together with outside partners, they could gain political capital. As one participant stated in a focus group:

“The old (political) party did nothing. They didn’t even clean the streets. The cleanliness of the town was gone. But as Haitians, we need to bring this back! We cannot live in our trash. Our carelessness about trash is the root of our poverty. We need to address it. If we do, we can help development. We can change the mindset of people. We have hope that this new group will improve the situation.”

As with many small urban towns in the Global South, garbage collection consisted of small payments to informal waste collectors, the regular burning of trash by households and a few acknowledged ‘dump sites’ (mostly the sides of various rivers). Larvae were common in waste sites; plastic bottles, vegetation, and other trash clogged the canal systems contributing to larval productivity. The municipal authorities had a team of 17 *vwaye* (cleaners*)* in-charge of cleaning the town. However, they were irregularly paid and concentrated on cleaning the streets, only really working before and after important holidays; they did not address the clogged canals—a major source of *Culex* larvae responsible for transmitting LF. The authorities paid them through taxes collected on construction sites, permits, and from the funds sent by the Ministry of Interior. 

We had cautiously hoped to partner with the municipal system, but efforts to move from the initial enthusiasm to concrete plans were met with avoidance and passive resistance from the three mayors, known as a ‘cartel’. Follow-up meetings were hard to arrange, agreed on details constantly changed and community workshops with the mayors were poorly attended. We needed their assistance for our initial community entry, recruitment of larval surveillance officers, recruitment of community mobilizers (described below) and cleaners, and assistance with organizing large events in the town. This was all shrugged off as ‘normal politics’. As one of our staff reflected in a project meeting:

“You move around, the mayor is not there. The door locked. You had a meeting but are told he has not been seen for days. His phone is off. You depend on him to help organize and plan. You have discussed this. But none of the cleaners will work without a push from the mayor…it’s complex. The mayors don’t do things that they should do, or say they will. (Ministry of Public Health and Population) is amazed we are even trying to work with them! Really they are amazed!”

Our efforts were at a standstill until we were introduced, by chance, to the third (woman) mayor. She was competent, punctual, and genuinely interesting in controlling mosquitoes. After much back-and-forth, we decided to abandon the idea of using the municipal cleaners and hired a team of health promoters to accompany the PKMs on their rounds, loosely based on the model used by the cholera response in Haiti. Using handheld megaphones (when appropriate) and other methods, the idea was that these teams—four teams of four people in each intervention *quartier*—would mobilize the community in environmental/household modification, promote personal protection measures, and increase knowledge about mosquito-borne diseases. This would include community mobilization for the cleaning of canals, the removing of riverine vegetation, filling-in ditches, removing tires and waste, and addressing various water container habitats (described more below). The focus was not on waste collection per se,but on habitats that we found to harbor larvae.

We wanted to select individuals with different skills: one good at community mobilization, one respected leader and two willing to ‘get dirty’ in the cleanup. Despite our knowledge about the challenges of the recruitment process, we were not yet in a position to select individuals from the community ourselves and had to rely on local leaders. The selection process got to the heart of the governance challenge in Haiti.

“Leaders say they are on board, but then they give you a bad person. Why? It’s so confusing! People will give folks that they know will not work. They give these people to show [these people who helped them politically] that they returned the favor…that’s it! They owe something to them.”

(Staff meeting.)

Part of this problem, which we eventually overcame, was a general sense from local leaders that we were not really serious about results and would not have the ‘courage’ to challenge them. As with the initial selection of the PKMs, many of our 16 promoters were not the best for the job. Over half were not prepared to work at all—the wife of one of the mayors, for example. Our relationship with the municipal authorities was fragile at the time, and we avoided a direct confrontation with them. Rather than confront them at this stage, we had to get some of the promoters to leave on their own accord, and still had to pay them for the time they (claimed) to have worked. This experience is common in Haiti, and reflects the ‘NGO-ization of services’:

“The influence of NGOs is so strong (in Haiti). NGOs do small work and say they did something big. They don’t believe in what they do and often don’t care. They only benefit themselves. There is no supervision on the ground, like you have. There is no accountability or pressure for results. It is all for a report. To say you did something. This attitude pressures people to be lazy and insincere. If no one works, then everyone is at the same level. So [at the community level] they oppress you if you work! It discourages people from working and puts people against each other.”

(Interview, senior government official, 2016.)

The need to maneuver between the cooptation of the project and relationship building with local authorities required careful negotiations. Again, it required “tactics” to avoid being ‘sabotaged’, and build trust and relational bonds. A good example of this involved our efforts to involve local churches in a community-based art competition: To register people in the competition, we spoke in front of different congregations and distributed sign-up sheets with church leaders. However, we found that one leader spread rumors to his church; that particular church did not participate. We discovered that this individual had wanted to be hired as a promoter but had been firmly refused by one of our field supervisors. As the local overseer of the election card system for the commune, he felt snubbed and was angry. We reflected on this with our supervisors. A more appropriate strategy, so the team decided, was to ‘maneuver’ in a more ambiguous fashion, to deflect responsibility for denying him the job and explain away any potential conflict. Point blank refusals often resulted in some local resistance and efforts to undermine our work, and there was a strong sense that, in Haiti, a small number of people, wanting to sabotage something, could easily succeed. As one senior Haitian health researcher, based in the US, commented to us:

“It is something I have noticed about (Haitians). We follow people. If someone is trying to spread rumors, it is hard to dispel them. People follow. Why? There is a suspicion of authority. It is maybe part of our history—against slavery, against authorities. But this can be very negative. It means that it is easy for a few people to derail something that is good for the country.”

(Interview, 2016.)

Another example of strategic maneuvering involved our interaction with the local *depute*, who represents the Commune in Port-au-Prince. During our second meeting, with the goal of obtaining in-kind support for waste collection and the cleaning of public spaces, we unfortunately used the word ‘project’ to describe our activities. The conversation quickly changed tones: “No! No! It is your project, so I will let you decide what to do”, was the response. Garbage collection is, after all, the responsibility of local government; they receive some funds from the Ministry of Interior, although it is unclear how often and how much. This drew attention to the pitfalls of the outsider status of the American team (author K.L.B.is referred to as *blanc* in Haiti), and the importance of local terminology in structuring notions of participation and purpose. 

With time, we managed to secure a contribution from the *depute*—new wheelbarrows, shovels, pitchforks, and brooms were provided for our community cleanup competition and a local NGO supplied food for participants. The local authorities also arranged subsequent cleanup campaigns in the town, especially for some of the major canals, and made signs telling residents to not throw garbage in the river. By involving the *depute*, we had other *deputes* and mayors in the Nord Department contact us and request that we expand the program to their localities. With the challenges of involving the mayors’ office, however, our efforts to address the systemic challenges of garbage in Plaine-du-Nord clearly required a more long-term plan.

### 3.3. Understanding Community Practices and Logics

In order to design our LSM approach in Plaine-du-Nord, we conducted formative social research on local understandings of mosquito ecology and disease as well as notions of self-efficacy, social organization and collective action. This played an important part in guiding our intervention plans and, as we will see, in mediating implementation and community response. 

Mosquitoes were described locally as ‘unbearable’, ‘vermin’, ‘invasive’ and ‘annoying’ in that they, as one woman stated, “make life even harder for us poor people…under God, they perpetuate violence and ruin. They are evil creatures”. Our household questionnaire (*n*=293) showed that mosquitoes were “very abundant” (97%), both inside the home (79%) and around people’s yards (72%). 84% of participants strongly agreed that diseases caused by mosquitoes were a major health priority (and willing to use time and effort to address them). Without any prompts, a free-listing question asked, “What diseases are caused by mosquitoes?” As shown in Table 2, malaria (86%), filariasis (72%), and chikungunya (56%) were the most commonly named. Interestingly, some people associated typhoid, cholera, and diarrhea/dysentery with mosquitoes, highlighting the strong association of risk factors for water-borne and mosquito-borne illnesses (39% of households in our HHQ did not have a latrine). A total of 66% (192/293) reported that they had suffered from a mosquito-borne illness in the last year, with the majority reporting chikungunya (49%) and Zika (21%) and some malaria and filariasis. This prevalence of chikungunya and Zika corresponds with the epidemic waves that swept into the Caribbean between 2014 and 2016.

Our qualitative research added some texture to this data. Here, the most widely cited illness caused by mosquitoes was ‘fever’ and/or ‘headaches’. These were often not linked to any particular disease, although they were occasionally synonymous for ‘malaria’ (many women reported that malaria was ‘incurable’ reflecting its strong local aetiological relationship with fevers). Due to the MDA campaigns, lymphatic filariasis (LF) was also widely known (associated with edema in the lower limbs and swelling, scrotal hernias,and other symptoms). MDA had a great deal of local support, and was considered one of the only consistent expressions of public health by the Haitian state (Despite this, LF was still considered a widely stigmatizing disease. Neighborhood songs about infected men with ‘gro grenn’ and ‘madougou’ (big testicles) were not uncommon). Although there was some basic knowledge about Zika virus, notably the risk for pregnant women and children, mostly acquired by radio, there had yet to be any reports of microcephaly in the region and people were unsure about it. Chikungunya virus, known locally as ‘paralysis’, was considered much more devastating. Whole families were reported to be sick for weeks during the 2014/2015 epidemic in the Caribbean, with many suffering what they believed were long-term sequelae like joint pain and aches—it had “spared no one”.

An intuitive relationship existed between the presence of breeding sites (canals and ditches, lakes, latrines, containers, and abandoned tires), mosquito abundance and human affliction. Larvae were described locally as *ti gounouche* (little frogs) or small worms, and frequently associated with stagnant and dirty water, reinforced by notions of (un)cleanliness. However, most people did not understand the larval development cycle—data from our HHQ shown in Table 3 shows a wide range of understandings of mosquito breeding sites, including under furniture (32%). This range of responses was also reflected in our qualitative data, although people tended to list many more potential breeding sites during focus groups. 

Discussions about community strategies to control larval habitats quickly veered into topics of poverty, lack of state services, disintegrating social cohesion, and the insufficient capacity of individual households to address what was considered a community and public problem. People did employ a number of (irregularly used) personal protective measures—mosquito nets, the use of smoke by burning garbage, ‘mosquito crème’ found at the local market, and other local repellants like eucalyptus and aloe vera, insecticide coils, motor oil applied into large habitats, lids and covers for water containers, chlorine and various types of screens and windows. Some neighborhood groups conducted periodic community cleanup, building on collective labor efforts done for roadwork and to maintain rivers and canals that periodically flooded during the rainy season—during our project, Plaine-du-Nord town was devastated by flooding in November 2016. We witnessed local cleanup efforts several times, although it was always very localized and not targeted to larval habitats. 

**Figure 2:**
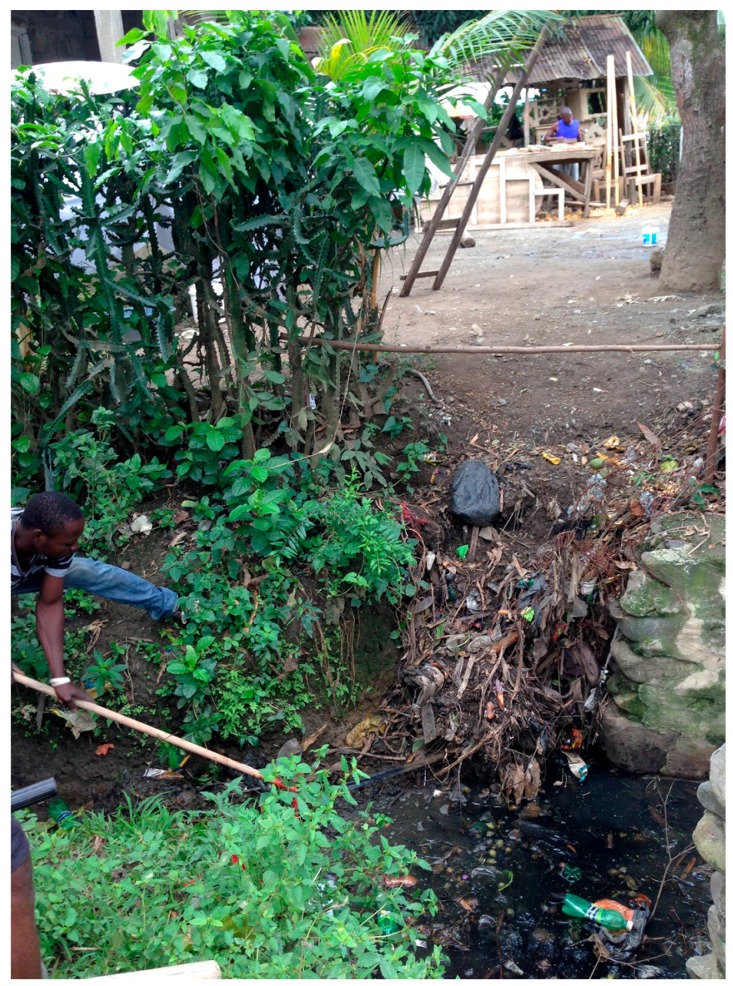
The problem of garbage and canals contributing to the proliferation of *Culex* mosquitoes, the vector of lymphatic filariasis in Haiti.

All of these efforts were consistently described locally as “only somewhat effective”; most people stressed the improbability of controlling mosquitoes, given the structural violence and poverty that surrounded them. Only 6% of people in our HHQ believed that they could effectively control mosquitoes. A great deal of these discussions focused on the topic of environmental sanitation and the inability of the state to engage in cleanup efforts. Figure 2 shows a typical situation, where a household (which has a middle-aged women suffering from lymphatic filariasis) continues to dump their household garbage in a street canal, blocking the drainage of the canal and contributing to the proliferation of *Culex* mosquitoes. People expressed the view that local leaders did not support cleanup efforts, for instance by providing materials, and that fellow community members were quick to be uncooperative, sometimes suspecting that someone was benefiting financially from their voluntary labor. 

Haiti, however, does have a long tradition of peasant associations and local democracy that could be used to support environmental cleanup. This includes the practice of *konbit* (collective agricultural labor), which is rarely still practiced in our study area. The *konbit* tradition has now morphed into *ranpono*. Whereas *konbit* was based on group solidarity, with meals provided by the owner of the land, *ranpono* involves daily waged labor. This shift was linked to a range of factors but, again, was dominated by a narrative of state and social disintegration: poor prices of agricultural products, bad soil fertility, migration of agriculturalists to the Dominican Republic to engage in waged labor, the large number of imported commodities, the cultural ‘globalization of the youth’, foreign political intervention, and a lack of trust in politicians. As one local leader stated in a planning workshop:

“I do not even remember the last time there was a *konbit* in the area. Even though a good planting of rice cannot be done without *konbit*. The Northern Plaine used to be the granary of Haiti but now the harvests are not profitable. One is obliged to consume imported rice (from the United States)…Misery has settled into our community. In the past, we all lived as a family in the community, when someone had a problem everyone lent him a hand. But no longer! People do not want groups; they want only money. I am used to hearing on the radio the Evangelist Joseph Taylor claim that ants are superior to humans because they have solidarity and union between them.”

There was a counter-narrative to this bleak prognosis, mostly voiced by youth groups and church leaders. This opinion stressed that, although things were hard, it was only through collective action and organization that Haiti could address its ‘diseases of under-development’ — mosquito-borne pathogens were viewed as a representation of this. As a solution, for example, people emphasized the need for NGOs and the government to invest in strengthening community solidarity for mosquito control by facilitating trainings, providing equipment and generating a sense of teamwork and leadership. In this view: *Vouloir c’est pouvoir* (If you want, you can). There was a long list of community groups in Plaine-du-Nord—youth groups, women groups, church groups, political groups, and income generation groups. Some of these occasionally did cleanup work, positioned against the notion that people could not improve the local environmental sanitation situation. Optimistic leaders believed that these efforts could be used to support mosquito control efforts, and could be done sustainably. As one youth leader stated in another planning workshop:

“I disagree with this idea (that people cannot work together to control mosquitoes). Haitians are very strong as soon as they want to get something. We won in 1804 (against the French)! Working together to control mosquitoes is very feasible. Yes, there is depravity here. Look at me! Come visit my house! But I want to learn. We all want to learn. We just need some material, some little help. We want to see the country advance. Let’s stop with this negative discussion. It is not true. We can address this issue. For a better future.”

### 3.4. Community Engagement for Larval Source Reduction

Our discussions with community members, MSPP and local people led us to design and trial a series of risk communication and community engagement interventions. The first of these included distributing a 20-page pamphlet, in both French and Creole, to each of the 1181 households in Plaine-du-Nord. This described the PKM initiative, the ecology of *Aedes*, *Culex*, and *Anopheles* mosquitoes, symptoms and consequences of filariasis, malaria, Zika, chikungunya, and dengue, and methods of prevention. Within this pamphlet, we included a phone number for community members to contact the project team, and described the conditions for a series of community-based competitions that we planned, in consultation with local leaders. 

The first series of competitions, each of which consisted of a small financial prize, included separate art competitions (on the theme of mosquito prevention) for poetry, painting, music, and drama. Our rationale was three-fold: (1) competitions were seen as an appropriate way to generate local interest in the project; (2) they would enable us to better identify influential individuals in the community that we could work with; and (3) it would create a repository of locally-generated health promotion material that could be used. In total, we conducted eight meetings with different church groups, six with youth groups, three with women’s groups, and two with other community groups to explain these competitions and to provide feedback on initial submissions. These were attended with a great deal of enthusiasm and interest. 

**Figure 3:**
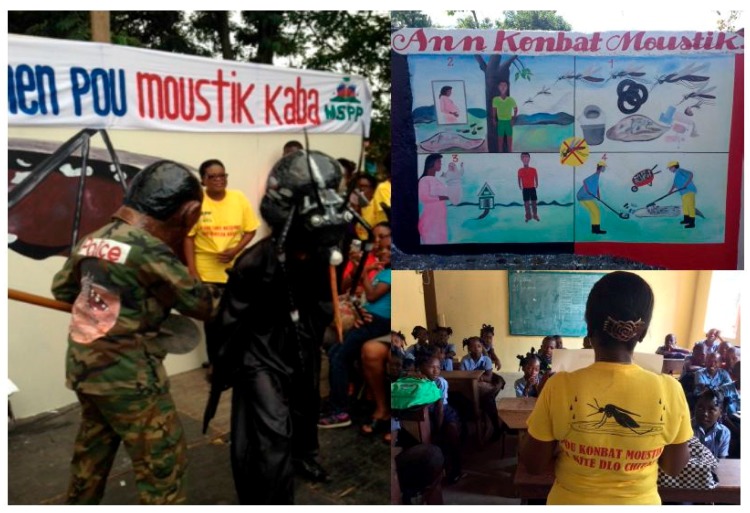
Mosquito mascot performance (**left**); a large painted mural from the art competition (**top right**); and a photo of the school-based program (**bottom right**).

We planned to involve and showcase these local artists on a large stage during the Festival St. Jacques, one of the largest and well-known voodoo festivals in Haiti, which happens to occur in Plaine-du-Nord town each July, attracting tens-of-thousands of pilgrims from throughout Haiti and the diaspora in the USA. With some co-financing from the MSPP HIV/AIDS program in the North Department, we hired a stage and DJ in the center of the then crowded town (our initial efforts to coordinate with the mayor’s office was unsuccessful), and had a large mosquito painted as a background. Over the course of the day, 24 different art groups performed. This included traditional and modern songs, including hip-hop and raga, paintings, poetry readings, and a series of dramas and comedy sketches, including the use of a mosquito mascot and a costume of a ‘mosquito policeman’ (see Figure 3). 

Directly after the festival, we began the second phase of our community competitions: an inter-community cleanup competition. The cleanup campaign used a slogan from the artists (‘*Nou tout se fre’* (we are all brothers), that drew on the tradition of *konbit*. Our intention was to use the competition to promote collaboration between the project, municipal government and community groups to address persistent habitats and the problem of garbage in the town. Using the PKMs, our newly hired 16 community promoters (described above) and some of the cleaners from the municipality, we spread the news of the competitions using loudspeakers and held a series of meetings in each *quartier* with community members, leaders, and those involved in the art-based competition. These meetings outlined the goals of the cleanup campaign, the financial reward for the ‘winning’ *quartier* and the methods of evaluation. Using the mosquito police and the promoters, we provided a list of the positive habitats that we had discovered during the four weeks of our larval surveillance and, with a camera, had the PKMs take photographs of these habitats to generate ‘before’ and ‘after’ pictures. T-shirts with slogans from the art competitions were distributed during the cleanup, and cleanup material from the municipal government and *depute* were used. After a few days, leaders from each *quartier* voiced their concern: despite the reward to the winning zone, people required a more immediate incentive. We decided to provide a small bag of rice to each person who participated for more than three hours a day, provided by a local NGO. Each group kept track of its daily effort and the number of people involved on a data sheet. 

The competition continued for two weeks. Two *quartiers* were very involved in the cleanup efforts, while two others quickly abandoned the effort, partially due to the fact that the chosen promoters and the PKMs in those zones were not very motivated, for reasons discussed above. The areas with the most dedicated leaders—Breda and Royan—were also those with the majority of artists that participated in the art competition and most engaged in the cleanup campaign. Some drew upon the songs and messaging material they developed to enroll support and volunteers. In the other areas (Plaine-du-Nord town and DuPerrier), it was clear that leadership was more diffused, there was less of a tradition of collective social service activity, and there was a more fractured sense of community. In these areas, people regularly told our staff that they wanted to be paid directly—as one leader told us, they “want money in their hands”. The *quartier* that won lived along a large river, which sometimes flooded and required regular maintenance by community members, and had a very active youth group. This group requested that during the two weeks of cleanup, instead of providing uncooked rice, we hire a cook to provide them with a lunch during their cleanup efforts. This was explained as a form of *konbit*. 

After the community cleanup competition ended, we then designed and implemented a school-based intervention. The goal of the school initiative was to use a *‘*citizen science’ approach to mobilize school children to act as ‘mosquito police’, or health change agents, in their home and community. Schools are established institutions in Haiti, and school children were very involved in the cleanup groups. In consultation with the directors and teachers of three local elementary schools, we designed a five-day education program and implemented it with over 300 students. This included: (1) the use of large poster-boards to guide education sessions on mosquito ecology, mosquito-borne diseases and prevention methods, which included pictures and graphics; (2) the use of plastic mosquito life cycle models (eggs, larva, pupa, and adult); (3) school-level microscopes; (4) comedy sketches using our mosquito mascot and policeman; (5) drawings and art; (6) cleanup of the school yard and surrounding areas; and (7) a homework assignment (with an identification guide for larvae and simplified surveillance data sheet) that included guiding students through the steps of larval surveillance with their family members. We found that students were very receptive to this intervention, curious to learn, and willing to cleanup larval habitats around their school and at home. Teachers were also impressed with the initiative, and stressed the fact that their classes rarely had the opportunity to be exposed to such didactic learning methods.

Overall, the art competitions, cleanup campaign, and school-based intervention stressed the importance of understanding local larval habitats, methods to address them, and the feasibility for community action. One concrete effect of this was an increase of community trust and willingness to allow our mosquito police to enter people’s homes for surveillance. It also allowed us to identify key individuals, as discussed above, who had the motivation and work ethic to be employed as our mosquito police when we increased our staff and began a new phase of surveillance and larval source management (LSM) in October 2016. It was also clear that regular and systematic environmental modification—many of the cleaned canals and other habitats quickly reverted back to their prior state—would be a major challenge using small incentives (prizes and food) and community events. Both activities were certainly important avenues to engage communities in LSM and increase knowledge about mosquito-borne diseases, but such efforts could not replace what was fundamentally lacking: functioning vector control and municipal waste collection systems with dedicated, trained, and paid staff. 

### 3.5. Community-Directed Larviciding and Environmental Cleanup

Our project activities did not occur in a vacuum. We were acutely aware of other stakeholders involved in filariasis and malaria control in Haiti, especially as we tried to integrate our efforts with the *Programme National de Contrôle de la Malaria* (PNCM). PNCM coordinates the LF MDA in Haiti and, with funding from the Global Fund, operates a network of department-level vector control teams, known as *brigadiers*. There are 12 teams throughout Haiti (each with four fieldworkers and one supervisor). These teams typically travel together in one vehicle, covering very large distances, for larviciding and the periodic (but irregular) use of truck-mounted and handheld thermal fogging. 

Within this context, we sought to test the feasibility of community-directed larviciding in our intervention *quartiers*, in collaboration with PNCM and MSPP. To do so, we engaged the *brigadiers* to train our mosquito police in the application of *Bacillus thuringiensis* subspecies *israelensis* (*Bti*) using backpack sprayers and hand application. A bacterial larvicide, *Bti* has become a favorite of large-scale LSM operations become it selectively targets only mosquito and blackfly larvae; it is environmentally friendly. The PNCM office, interested in piloting a new approach, provided the necessary equipment and materials. 

Our qualitative research with the *brigadier* teams showed a number of salient operational gaps. It was not always clear why or how the teams targeted specific areas, and little data to show how effective their efforts were being. Their major focus was *Anopheles albimanus*, the primary malaria vector, leaving a gap in the control of *Culex* and *Aedes* mosquitoes. A number of challenges were repeatedly highlighted in our interviews, focus groups and participant observation: lack of staff, low capacity, poor work morale (and accountability), periodic lack of transport (and fuel) and limited ability to effectively engage communities (mostly left to periodic radio messaging and the occasional poster). All of this was compounded by a general gap in middle-level management. 

Our relationship with the local brigade team evolved with time. At first, we had sought to fully engage them, consulting them on site locations, study design and community approaches. However, there was a clear reluctance on their part, and a suspicion that we had come to monitor them and perhaps critically evaluate their activities. They were also busy; our attempt to have them conduct weekly quality checks did not work, despite our substantial effort to do so. ‘Small incentives’ for activities were a continuous negotiation that, beyond the occasional phone card or small stipend, we could not afford. However, once we ‘hired’ them to train the mosquito police in larvicide application, the *brigadiers* became much more interested. They enjoyed the idea of managing a group of ‘sub-*brigadiers’* and our relationship improved, especially as we explained that our long-term goal was to create networks of such agents to work under their supervision. As the *brigadiers* visited our field sites, they became impressed with the scope of our work. Officially, there were only eight larval habitats in our four intervention *quartiers* in Plaine-du-Nord that the *brigadiers* registered as part of the more than 400 documented habitats in the North Department. They visited these sites every few months. 

Towards the end of our pilot project, therefore, we arrived at a ‘model’ that would integrate community-directed larvicide with environmental modification, risk communication and community engagement. In order to accomplish this, we first had each mosquito police (PKM) conduct larval surveillance for the first three days of the week and a list was then generated of the positive habitats. PKMs, supervisors, *brigadiers*, and local residents would then address them. To circumvent the problem of incentives for community participation and the issues with the municipal authorities, we decided to hire eight day-laborers to carry out physically demanding cleanup activities, like canal cleanup, under the direction of the team. 

Our entomology data showed a range of important habitats for culicine (*Aedes* and *Culex*) larvae: street canals, tires, plastic drums, puddles/tire tracks, containers, water tanks, and artificial groundholes. Different habitat types were found for anopheline larvae, mostly with vegetation. While we will present our entomological data in another publication, this data helped us target our cleanup and larvicide activity to the most important habitats. It is worth noting here, however, some of the challenges with designing larval control pilots and causative attribution. As this was a pilot intervention focused on testing a range of community mobilization strategies, by the time we had built the necessary local relationships to arrive at our integrated model, we had effectively run out of money. Time was limited. Larval data, however, need to be collected over an extended period of time; ecological, climatic, and social factors are embedded in local landscapes and each site has unique characteristics. As a dynamic system, larval sites can also become, as one senior MSPP staff told us, a game of ‘ping-pong’: control of certain habitats can push female adults to oviposit in others. Habitat abundance and frequency can change with seasons. Mosquitoes can also fly between intervention and non-intervention sites. Rice fields, large lagoons, and other habitats can be difficult to access. As the mantra goes, it is important to target control activities to the few, fixed, and findable habitats to be most (cost) effective; but determining these data takes time.

## 4. Discussion and Conclusions

This paper has reflected, ethnographically, on the social relationships, negotiations, and maneuvering involved in designing and implementing a community-directed mosquito control pilot project in a small town in northern Haiti. Other forthcoming papers will present our entomological data and de-worming (MDA) drug uptake data for lymphatic filariasis. Here, our focus was to explore a more nuanced, and often overlooked, social and political perspective on the implementation process, in the context of poverty, resource limitations, and a general lack of effective state services and governance. A large body of applied social science research on infectious disease has shown that better community engagement and behavior change is not only about designing better messages, but more about building trust, social learning, and the ability to iteratively adapt plans based on feedback from community members and field teams [1]. Our approach here assumed that, as Bardosh [36] noted in Uganda, global health interventions are evolving ‘social experiments’. Dominant models and plans require flexibility. Power and politics need to be understood and engaged [37]; and planners and practitioners need to recognize that mistakes need to be made for learning to take place. 

The *Polisye Kont Moustik* (PKM) approach, as we developed it, required a great deal of culturally-sensitive ‘tactics’ to avoid the various forms of ‘sabotage’ that we encountered in the field. These are soft skills. Ultimately, through cycles of formal research, feedback, meetings, discussions, brainstorming, and tacit learning, the PKM team developed not only specific education and social engagement strategies but also, perhaps more importantly, the relational capital needed for a viable approach. Community-directed mosquito control, centered on strong local partnerships and participation, developed through various social processes: from our mosquito marker, project pamphlet, written and practical tests to evaluate new potential recruits, repeated community and local leader workshops and meetings, the use of ‘police’ as a metaphor to guide programmatic work, the implementation of our community competitions, and the testing of different management systems (such as sign-in and out sheets) to supervise field workers. Cultural competence, specifically the decades of experience of co-author LJ as a Ministry of Public Health and Population (MSPP) program planner, enabled us to maneuver the expectations and interests of different community members, around the recruitment process and the involvement of community members and municipal authorities, for example. 

However, we struggled in important ways, conceptually and practically, with where our approach fit within the continuum of ‘top-down’ (technocratic) or ‘bottom-up’ (participatory) health promotion [38]. Our original vision was to develop an approach led by ‘the community’. But what does this mean? The PKM approach involved substantial engagement with a wide variety of community members, as we have described throughout this paper; specific strategies, educational messages, and field activities were developed and led by local leaders and our staff, people who were involved in planning the project at multiple stages. However, certain predetermined goals and technical expertise guided these discussions. The process was iterative and dynamic, moving from learning with and consulting community members, considering budgets and our team capacity, and then reflecting on our project objectives and the wider context of the vector control landscape in Haiti. We could say that, in one sense, this led us to ‘police’ our approach—activities, in the end, were directed by the technical team. What would a more participatory approach look like? In important ways, the focus on controlling mosquitoes and showing that our approach worked narrowed the possibilities for action. The emphasis on quantitative results, to measure entomological ‘impact’ and apply for a Phase 2 BMGF grant, led us to prioritize a more technical system. For example, we originally intended to have local ‘committees’, made up of selected formal and informal leaders, design, plan, and guide all field activities in each *quartier*, based on real-time data and some direct resource support. The complexities of getting the technical work up and running—the PKM system—and juggling the variety of our activities meant that this effort was pushed to the side. 

Technical details are challenging for vector control. Questions remain in Haiti regarding the geographic scope of LSM and the operations/management of the current *brigadier* team. In a recent Cochrane review of LSM for malaria control, Tusting et al. [39] stressed the often-repeated maxim: that for LSM to be successful, a sufficient proportion of larval habitats should be targeted. In Haiti, even baseline entomological intervention data is sparse and needs to be updated to inform decision-making [33]. The current larvicide activities of the *brigadier* teams are not validated against parasitological or entomological indicators, nor are the activities directed to households of reported and infected malaria patients. A lack of linkages between intervention, evidentiary evaluation, and targeting stymies scale up, and scale-out of this type of approach. This needs to be addressed, preferably while incorporating elements of the community-based PKM approach discussed in this paper. Historical examples of LSM using drainage, ditching, and larviciding have been used effectively in the past in Haiti, and should be reconsidered [33,40]. In the context of a low transmission setting, however, geographical scope, staff structures, and the necessary frequency of activities remain an open question. 

A recent large-scale case-control study in Port-au-Prince found no evidence that mass ITN distribution, in 2012, reduced clinical malaria cases, and advocated for new alternative strategies, like MDA [41]. As MDA strategies gain more traction for malaria more generally, there has been a renewed interest in community engagement [42], especially where malaria is responsible for only a small portion of febrile illnesses and where high levels of coverage (>85%) are required over two or more rounds of treatment with drugs like sulfadoxine/pyrimethamine. Many local people strongly associate malaria with mosquitoes (without differentiating species) and believe that malaria control needs to have a vector control component, which might help facilitate stronger community buy-in for MDA in Haiti [43]. Community dialogue (not only ‘educating at’ people), in schools, churches, municipal offices and other local settings, will be key. 

Of course, any malaria MDA strategy could learn from the decades of experience of the LF program [32]. Even here, however, a more integrated approach, such as door-to-door MDA delivery and the cleanup/larviciding of canals and other major *Culex* breeding sites (as tested in this pilot project) may be needed to effectively eliminate the filarial parasite in certain foci [21,23,24]. A number of continued hotspots of residual LF transmission, many in urban areas, will benefit from this approach as part of a new USAID-funded Zika initiative, which should be harnessed to have added benefit for LF elimination.

Some scholars and practitioners question the feasibility of LSM in resource-poor settings like Haiti [18], and others optimistically point to a new, expanding technological toolbox of mosquito control techniques: dominant lethal genes and endosymbiotic (*Wolbachia*) bacteria for *Aedes* control [44]. Our pilot study showed that social engagement, community education, and an effective community-directed ‘ground-game’ can be mounted in Haiti for the integrated control of mosquito-borne diseases, albeit further research (some in preparation, others planned) are needed on behavior change, community involvement, entomological impact, and sustainability issues. Using a range of engagement/education strategies helps strengthen behavior change and knowledge uptake, while building partnerships with municipal authorities and formal and informal community leaders facilitates and reinforces the process [45]. As we have endeavored to show here, the better integration of formative research, social learning, and community participation ultimately assists in navigating important social and political contexts, and should play an important role in future malaria and lymphatic filariasis elimination in Haiti and elsewhere. 

## Figures and Tables

**Table 1:** Larval Surveillance Mapping System.

	*Quartier*	Number of Blocks	Number of Places	Houses	People
Intervention	Plaine-du-Nord	47	516	455	1347
	Breda	49	506	255	1083
	Royan	30	343	236	817
	Duperier	34	400	235	1010
Control	Carrefour des Pierre	40	379	339	1133
	Suisse	44	583	185	699
	Dimini	41	500	527	2434
	Haut du Cap	34	340	277	718
	**Total**	319	3567	2509	9241

**Table 2:** Knowledge and Experience of Disease.

	What Diseases are Caused by Mosquitoes?	Self-Reported Illness Episode in the Last Year
Malaria	86% (253/293)	12% (35/293)
Filariasis	72% (210/293)	1% (3/293)
Chikungunya	56% (165/293)	49% (144/293)
Typhoid	14% (40/293)	----
Cholera	16% (47/293)	9% (26/293)
Severe diarrhea/dysentery	8% (23/293)	43% (126/293)
Zika	27% (79/293)	21% (62/293)
Dengue	11% (31/293)	----
None/unsure	3% (2/293)	----

**Table 3:** Community Identification of Mosquito Breeding Sites.

Where do Mosquitoes Lay Their Eggs?	
Stagnant water	62% (185/293)
Inside the house (under the bed, table and dressers)	32% (95/293)
In the canals	38% (111/293)
Everywhere	14% (40/293)
In the forest	11% (31/293)
In the river	10% (28/293)
In agricultural areas	15% (43/293)
Other (near animals, the street, garbage, in toilets, etc.)	7% (21/293)
I don’t know	34% (100/293)
